# Long-Term Implicit Epigenetic Stress Information in the Enteric Nervous System and its Contribution to Developing and Perpetuating IBS

**DOI:** 10.2174/1570159X22666240507095700

**Published:** 2024-05-09

**Authors:** Császár-Nagy Noemi, Petr Bob, István Bókkon

**Affiliations:** 1 National University of Public Services, H-1083 Budapest, Hungary;; 2 Psychosomatic Outpatient Clinics, H-1037 Budapest, Hungary;; 3 Center for Neuropsychiatric Research of Traumatic Stress, Department of Psychiatry & UHSL, First Faculty of Medicine, and Department of Psychiatry, Faculty of Medicine Pilsen, Charles University, CZ-12108 Prague, Czechia;; 4 Neuroscience and Consciousness Research Department, Vision Research Institute, Lowell, MA 01854 USA

**Keywords:** ENS, Implicit epigenetic long-term memory, IBS, microbiota-gut-brain axis, stress, short-chain fatty acids

## Abstract

Psychiatric and mood disorders may play an important role in the development and persistence of irritable bowel syndrome (IBS). Previously, we hypothesized that stress-induced implicit memories may persist throughout life *via* epigenetic processes in the enteric nervous system (ENS), independent of the central nervous system (CNS). These epigenetic memories in the ENS may contribute to developing and perpetuating IBS. Here, we further elaborate on our earlier hypothesis. That is, during pregnancy, maternal prenatal stresses perturb the HPA axis and increase circulating cortisol levels, which can affect the maternal gut microbiota. Maternal cortisol can cross the placental barrier and increase cortisol-circulating levels in the fetus. This leads to dysregulation of the HPA axis, affecting the gut microbiota, microbial metabolites, and intestinal permeability in the fetus. Microbial metabolites, such as short-chain fatty acids (which also regulate the development of fetal ENS), can modulate a range of diseases by inducing epigenetic changes. These mentioned processes suggest that stress-related, implicit, long-term epigenetic memories may be programmed into the fetal ENS during pregnancy. Subsequently, this implicit epigenetic stress information from the fetal ENS could be conveyed to the CNS through the bidirectional microbiota-gut-brain axis (MGBA), leading to perturbed functional connectivity among various brain networks and the dysregulation of affective and pain processes.

## 1 INTRODUCTION

The exact cause of irritable bowel syndrome (IBS) is still unknown. However, most experts believe that it is related to digestive problems and increased sensitivity of the gastrointestinal tract. There is growing evidence that psychological factors play an important role in the development of IBS [1-3]. Evolution has endowed the gastrointestinal tract (GT) with its dedicated nervous system. The enteric nervous system (ENS) serves as a central control center for regulating the digestive and immune functions of the GT [4]. In mammals, the ENS plays an important role in controlling gut motility and secretion, local blood flow, and the intestinal immune system, among other functions. From an evolutionary standpoint, the ENS predates the development of the central nervous system (CNS) [5, 6]. The ENS developed earlier than and independently of the CNS and, therefore, could be considered the “first brain” [7].

Previously, we hypothesized [8] that stress-induced implicit memories can endure for an individual’s life through epigenetic processes within the ENS. These memories may play important roles in the development and persistence of IBS. This nonconscious (implicit) epigenetic-stress information within the ENS could be transmitted to the CNS *via* the microbiota-gut-brain axis (MGBA), thereby disrupting functional connectivity among multiple brain networks and dysregulating affective and pain processes, as evidenced by various neuroimaging studies [9-14]. However, the CNS must manage this nonconscious stress information from the ENS, leading to the activation of stress-response systems, such as the hypothalamic-pituitary-adrenal (HPA) axis and the immune system, and affecting gut processes. This creates a self-perpetuating “vicious cycle.”

Herein, we further develop our previous hypothesis [8] and present a possible simple mechanism by which maternal stress may produce stress-induced long-term epigenetic implicit memories (SLEIM) in the ENS of the fetus.

## 2 MICROBIOTA-GUT-BRAIN AXIS: POSSIBLE RELATIONSHIPS BETWEEN MENTAL DISORDERS AND INFLAMMATORY DISEASES LINKED TO THE GASTROINTESTINAL TRACT

The MGBA is a specific bidirectional communication system between the CNS and the ENS. It connects the emotional and cognitive centers of the brain with peripheral intestinal functions. The MGBA consists of the CNS, the autonomic nervous system (ANS) - which includes the sympathetic nervous system (SNS), the parasympathetic nervous system (PNS), and the ENS - the vagus nerve, the neuroendocrine system, neuroimmune systems, the HPA axis, and the gut microbiota and its metabolites [15, 16]. The bidirectional communication in the MGBA occurs through various pathways, including the vagus nerve, the immune system, neuroendocrine pathways, and bacteria-derived metabolites. Since the gut microbiome is an integral part of the gut-brain axis (GBA), it is more appropriate to use the term microbiota-gut-brain axis (MGBA) than the term gut-brain axis (GBA).

The MGBA, through multiple pathways and microbiota-derived metabolites, potentially influences a variety of functions, including memory, behavior, learning, neurotransmission, synaptic plasticity, stem-cell proliferation, brain development, mental and neurodegenerative disorders, the endocrine system, and the immune system [16, 17-22]. Diverse factors, such as drugs, antibiotics, pathogens, and psychological stress, can disrupt the microbiota, leading to dysbiosis (perturbations in the intestinal microbiota) and impairing communication within the MGBA [23, 24]. The MGBA could contribute to the development of numerous intestinal and extraintestinal diseases, among which are IBS, FM, chronic pain, stroke, lung disease, obesity, type 2 diabetes, insulin resistance, psoriasis, cancer, celiac disease, metabolic syndrome, and nervous system disorders, but the evidence is often limited to animal studies or correlations [25-28]. This axis has been shown to influence neurotransmission and the behaviors that are often associated with neuropsychiatric conditions [29, 30]. There is, in fact, some evidence that the MGBA plays some roles in brain homeostasis and may also influence the pathogenesis of certain disorders, including Parkinson’s disease, Alzheimer’s disease, multiple sclerosis, autism spectrum disorder, and major depressive disorder [31, 32].

In addition, the gut-associated lymphoid tissue (GALT) is the largest immune organ in the body that is located in the gut. GALT is an important component in sensing and responding to the microbial environment and contributes significantly to MGBA processes [33]. In humans, GALT comprises secretory lymphoid aggregates known as Peyer’s patches that sense and fight constant exposure to pathogens and infectious agents. Crucial to the functions of the Peyer’s patches is their communication with the ENS. Crosstalk between these tissues contributes to the MGBA, which influences mood and behavior as well as the homeostasis of neurological development and disease [33].

Psychological stress can impact inflammatory comorbidities and contribute to immune activations [34, 35]. Through the MGBA, mental stress could impact various inflammatory disorders associated with the GT [35, 36]. Recent findings indicate that it is not uncommon for patients with depression, for instance, to have multiple inflammatory comorbidities [37-39]. These processes may have significant implications for the complex relationships between mental disorders and inflammatory diseases associated with the gut [35, 40, 41]. The microbiota in the gut, along with related lymphoid tissue and glial cells, primarily mediates these multidirectional interactions within the MGBA [22, 39, 41].

Intestinal sensitivity, motility, secretion, and permeability are all significantly impacted by psychological stress, and the underlying mechanisms are closely linked to changes in the central nervous system, peripheral neurons, gastrointestinal microbiota, and mucosal immune activation [42]. It seems that MGBA can be the source of the compelling connection between psychological stress and IBS [42]. In addition, MGBA can also have an essential role in inflammatory bowel diseases (IBDs), as it increases the inflammatory response in the CNS and further contributes to anxiety- and depression-like behavioral comorbidities [43].

## 3 THE VAGUS NERVE: A KEY COMPONENT OF THE MGBA

The vagus nerve (VN) is a part of the autonomic nervous system that regulates numerous involuntary body functions to maintain homeostasis [44]. The VN is the longest cranial nerve, extending from the brain through the thorax to the abdomen, and can regulate blood pressure, heart rate, respiration, digestion, and the immune response [45]. The VN is part of the parasympathetic and sensory nervous systems that have a direct connection between the brain and the ENS and is the main afferent pathway from the abdomen to the brain [46]. The VN can sense alterations in the gastrointestinal lumen through interaction with other cell types. Enteroendocrine cells (EECs) are epithelial cells in the gut that directly interact with the VN [47]. The VN is considered a key component of the MGBA, which connects the gut and the brain bidirectionally [48]. Microorganisms in the gut can activate the VG, and this activation plays a critical role in mediating effects on the brain and behavior. VG plays a key role in the MGBA because vagotomy blocks CNS-related behaviors in rodents [49, 50]. In addition, even in the absence of overt inflammation, the VN can discriminate between non-pathogenic and pathogenic bacteria, and vagal pathways convey signals that can produce both anxiogenic and anxiolytic effects, depending on the nature of the stimulus [46].

## 4 PHARMACEUTICAL DRUGS COULD INDUCE DYSBIOSIS AND PERTURB MGBA

About 15000 pharmaceutical drugs have been developed so far [51]. A few of these medications are taken for long-term health issues like diabetes, high blood pressure, cancer, and arthritis. The most common drugs used are antidiabetic and cardiovascular drugs, pain relievers, proton pump inhibitors, and antidepressants [51].

Numerous studies found that pharmaceutical drugs such as antibiotics, antidepressants, statins, non-steroidal anti-inflammatory drugs, and other drugs could induce dysbiosis that influences and perturbs MGBA [51-53]. Antidiabetics, proton pump inhibitors, nonsteroidal anti-inflammatory drugs, and atypical antipsychotics were also associated with perturbations of microbiome composition [54]. Drug-induced dysbiosis may influence brain diseases through MGBA.

The activation of µ-opioid receptors (MORs) by agonists could induce dysbiosis, which is associated with the development of opioid analgesic tolerance, opioid-induced hyperalgesia, and the progression of chronic pain conditions such as neuropathic pain [55]. A recent study by Bernabè *et al.* [56] reinforced the notion that antibiotic-induced gastrointestinal dysmotility directly correlates with gut dysbiosis as well as structural and functional damage to the ENS. Statins are used to regulate serum cholesterol and reduce the risk of heart disease. Statin therapy produces gut dysbiosis in mice through a pregnane X receptor-dependent mechanism [57].

Individual reactions to a given medication can differ significantly in terms of both toxicity and efficacy. The gut microbiome, the host, and medications interact in a variety of intricate ways. In the future, however, changes in the gut flora should be taken into account when assessing the safety of medications [58].

## 5 IRRITABLE BOWEL SYNDROME

IBS is a multifactored disease that involves both central and peripheral pathophysiological mechanisms [59]. It is one of the most commonly diagnosed chronic functional gastrointestinal diseases worldwide, affecting 9-23% of the population [60, 61]. IBS is characterized by abdominal pain, bloating, and changes in bowel habits, such as diarrhea, constipation, or alternating constipation and diarrhea [62]. Additionally, IBS patients often experience dyspepsia, dysphagia, non-cardiac chest pain, and nausea [63]. Women are more frequently affected by IBS than are men [64].

Current treatments for IBS span a variety of approaches, including pharmacological interventions, psychotherapy, dietary modifications, microbiota transplantation, and complementary and alternative therapies [65-69]. However, it is important to note that these treatments aim to alleviate symptoms rather than provide a complete cure for IBS. Staudacher *et al.* [70] reviewed the epidemiology and impact of IBS, depression, and anxiety and considered the shared pathophysiology among these conditions. The authors recommended an integrated (holistic) approach and treatment for IBS. Explicitly, their suggested treatments for managing individuals with IBS and co-occurring symptoms of anxiety or depression are: 1. Medical treatments (low-dose tricyclic antidepressants or selective serotonin reuptake inhibitors (SSRI)); 2. Dietary treatments (Mediterranean diet); 3. Psychological treatments (Brain-gut behavior therapy, such as cognitive behavioral therapy and hypnotherapy).

Although the etiology of IBS is multifaceted and not yet fully understood, various factors, including genetics, epigenetics, stress-related effects on nervous and endocrine systems, immune dysregulation, dysbiosis, altered gastrointestinal motility, visceral hypersensitivity, post-infectious reactivity, food sensitivity, and carbohydrate malabsorption have all been implicated in the pathophysiology of IBS [63, 71-73]. Corticotropin-releasing-hormone-dependent dysregulation of the MGBA is involved in IBS, suggesting that IBS may be a disorder characterized by disturbances in brain-gut interactions and stress response systems, such as the HPA and ANS [74].

There is a growing body of evidence implicating psychiatric and mood disorders, including anxiety, depression, bipolar disorder, suicidal attempts, and eating disorders, in the development and maintenance of IBS [75-81]. Approximately 50-90% of IBS patients have comorbid psychiatric conditions, with anxiety disorders and depression being the most prevalent [82]. Maternal prenatal and postnatal anxiety and depression, maternal separation, and early adverse life events, such as physical and sexual abuse or emotional trauma, have been found to play a role in the development of functional gastrointestinal diseases and IBS [66-71]. Additionally, studies have found familial clusters of IBS, suggesting the possibility of an inherited component across generations [83-88].

## 6 INFLAMMATION MAY PLAY A PATHOGENIC ROLE IN IBS

Up to now, the underlying pathophysiology of IBS remains incompletely understood [89]. As mentioned in the previous section, the pathogenesis of IBS is multifactorial and complex, involving numerous factors [90, 91]. The digestive tract is a complicated structure that encompasses a single layer of epithelial cells, a mucosal barrier, the host mucosal immune system, and microbes [92]. The invasion and proliferation of pathogenic species, as well as the disruption of the immune system and mucosal barrier homeostasis, can be caused by dysbiosis that is due to harmful biopsychosocial factors [93]. In addition to helping to preserve intestinal homeostasis and reduce inflammation, the gut microbiota collaborates to influence host immunity. The host receives benefits from the gut microbiota in many forms, such as aiding in digesting, producing nutrients, detoxifying the body, defending against infections, and controlling the immune system [94].

Studies suggest that perturbed microbiota by various factors may be a key reason for the development of low-grade inflammation. Ng *et al.* [95] proposed the role of mucosal inflammation in the disease process of IBS. Recent data have found the pivotal role of intestinal microbiota in mucosal immunity [96]. However, despite the fact that conflicting findings are regularly reported, there is mounting evidence linking IBS patients to low-grade inflammation and innate immune system malfunction [95, 97, 98]. In order to provide a more precise picture of immunological activity, recent research has looked at mucosal samples. These investigations have shown that IBS patients have increased levels of proinflammatory cytokines and immunocytes infiltrating the mucosa [97].

IBS is linked to leaky bowels, in which the integrity of the gut blood barrier is perturbed, causing gut contents such as immune cells and microbiota to enter the bloodstream and produce low-grade systemic inflammation [99]. This increased level of inflammation-associated cytokines in circulation may affect all organs, including the brain. Central inflammation in the brain is associated with neurodegenerative diseases such as Alzheimer's disease, Parkinson's disease, and multiple sclerosis, as well as with neuropsychiatric disorders, specifically depression and anxiety [100-103].

Numerous research studies have investigated the potential implications of low-grade inflammation in the intestinal mucosa and systemic circulation, as well as the role that disruption of the innate immune response plays in the pathophysiology of IBS. Some evidence supports a primarily inflammatory mechanism that affects the intestinal mucosa locally and possibly systemically [97]. Nevertheless, contradictory findings have emerged from a large number of studies that have concentrated on particular cell types, cytokines, or pathogen recognition receptors. As a result, the precise function of immune cells and mediators is still unknown, and the underlying mechanisms remain obscure.

## 7 THE ENTERIC NERVOUS SYSTEM: DEVELOPMENT, STRUCTURE AND THE ROLE OF LONG-TERM EPIGENETIC IMPLICIT INFORMATION

As mentioned above, the ANS consists of three main components: the SNS, the PNS, and the ENS. Its primary role is to regulate the body’s unconscious or involuntary processes, including heart rate, blood pressure, respiration, digestion, and sexual arousal [104]. The ENS has a central role as an integrating hub for controlling gastrointestinal physiology [105].

The ENS includes millions of neurons and glial cells derived from neural crest cells (NCCs), which migrate to the gut and then colonize the entire length of the gastrointestinal tract and are organized into interconnected ganglia embedded within the gut wall [106, 107]. There are structural, functional, and chemical similarities between the ENS and the CNS [108]. The development of the ENS requires extensive cell migration, regulated cell proliferation, differentiation, neurite outgrowth, and interconnected neuronal networks [109]. The ENS uses more than 30 neurotransmitters, similar to the brain [110], including serotonin, dopamine, norepinephrine, and epinephrine, as well as amino acid neurotransmitters such as gamma-aminobutyric acid (GABA), glycine, glutamate, histamine, and acetylcholine [111].

The ENS is a highly intricate and independent nervous system that develops before and separately from the CNS [7, 112]. Neural circuits within the ENS are capable of performing localized and autonomous functions. Although the ENS and CNS can communicate bidirectionally through the MGBA, the ENS also possesses the ability to function independently. Moreover, evidence suggests that the ENS may have the capacity for learning and memory [113-115]. The ENS has been proposed to play a role in memorization and implicit learning, functioning akin to a “little brain” or a “second brain” within the gut [114].

### 7.1 Epigenetic Regulations in the Development and Functioning of the ENS

Epigenetic regulations, such as DNA methylation, histone modification, and microRNA regulation, can play a crucial role in the development and functioning of the ENS [116-120]. Numerous congenital and adult-onset gastrointestinal disorders are caused by defects in the development and function of the ENS, including Hirschsprung disease (HSCR), Esophageal Achalasia, Chronic Constipation, and Gastroesophageal Reflux Disease [121]. Hirschsprung disease (HSCR), which is the most prevalent ENS developmental defect, is brought on by aberrant NCC migration, proliferation, differentiation, or survival, which disrupts ENS development [122].

ENS development has been studied in various embryologic model systems, including zebrafish, avians, and rodents [123]. A relevant model for comprehending the development of vertebrate ENS is the zebrafish [124]. Although having a simpler architecture, the ENS of zebrafish is comparable to that of its mammalian counterpart.

Various genes involved in NCCs differentiation during ENS development have been identified, mostly from animal studies. Early enteric neuron growth from vagal and NCCs during ENS development is regulated by genetic, epigenetic, and signaling processes [118]. For example, in zebrafish experiments, Ganz *et al.* [125] found that ENS phenotypes detected within double mutants for *uhrf1* and *dnmt1* are not more severe than those of the single mutants. The authors suggested that Uhrf1 and Dnmt1 cooperate and that DNA methylation as a whole is required for proper ENS development. Thus, *uhrf1* and *dnmt1* could be potential new Hirschsprung disease candidates.

DNA methylation is also linked to ENS development because enteric progenitor cells (EPCs) have decreased DNA methyltransferases (DNMT catalyzes the transfer of methyl groups to specific CpG sites in DNA) expression in HSCR patients compared to controls, and some HSCR patients have presumed pathogenic missense mutations in Dnmt3b [122].

Polycomb repressive complexes 1 and 2 (PRC1 and PRC2) are key epigenetic regulators through histone modification of gene expression that are involved in almost all developmental stages. Feng and Sun [126] suggested that the *rnf2* gene (which encodes Ring1b, the enzymatic component of the PRC1 complex) plays an important role in the migration and differentiation of neural precursor cells, but loss of *rnf2* gene function produces abnormal development of the ENS and CNS in zebrafish.

## 8 METABOLIC COMPOUNDS PRODUCED BY GUT MICROBES

The appropriate composition of the gut microbiota and the metabolic compounds produced by gut microbes are key determinants of human health and disease. Countless metabolic compounds are produced by gut microbes including secondary bile acids; lipids; amino acids (including tyrosine, tryptophan, leucine, valine, and isoleucine); neurotransmitters (including serotonin, γ-aminobutyric acid (GABA), acetylcholine, and noradrenaline); vitamins as C, K, and B-complex; gases (including hydrogen (H_2_), methane (CH_4_), carbon dioxide (CO_2_), hydrogen sulfide (H2S), and nitric oxide (NO)); short-chain fatty acids (SCFAs) (including acetate, propionate, and butyrate) [127-129]. Perturbed production of these metabolites can produce various diseases, including metabolic diseases, cardiovascular diseases, gastrointestinal diseases, neurodegenerative and mental diseases, and cancer [127]. Here, we do not aim to delve into more details to discuss the roles of the metabolites of the gut microbiome. According to Swer *et al.* [130], “The exact underlying mechanisms of most of these metabolites are not understood; however, evidence of their effects on the brain has been reported in multiple studies”.

## 9 MATERNAL PRENATAL STRESS

Human and animal studies have demonstrated that maternal prenatal stress can alter the maternal gut microbiota and influence the composition of the microbiome of the offspring [131, 132]. During pregnancy, various factors, such as maternal obesity, diet, maternal stress, depression, infections, antidepressants, and antibiotics, can disrupt the maternal microbiota, affecting the development of fetal MGBA [133, 134].

The prevalence of antenatal depression (also referred to as prenatal or perinatal depression) or anxiety (or both) ranges from 8% to 30% [135]; there is wide variation among estimates, likely due to differences in measurement methods and sociocultural factors in individual studies and cultures [132]. Prenatal exposure to maternal stress is associated with mental and behavioral problems in children and later in life [136, 137].

Maternal psychological stress during pregnancy can perturb the HPA axis, thereby increasing circulating cortisol levels, which can affect the maternal gut microbiota [138]. Maternal cortisol can cross the placental barrier, increase circulating levels in the fetus, and lead to dysregulation of the HPA axis, thereby affecting the gut microbiota, microbial metabolites, and intestinal permeability, among other processes [139-141]. Maternal psychological stress is also associated with intestinal dysbiosis in the offspring and can affect the permeability and integrity of the fetal gut [142]. This stress-induced maternal dysbiotic microbiota in newborns shapes the stress responses of the offspring into adulthood through dysregulation of the HPA axis [137, 143, 144]. SCFAs are the major metabolites produced by the microbiota [145] that can control the cortisol response to psychosocial stress [146].

The metabolites of the gut microbiome can affect various diseases by inducing epigenetic changes through DNA methylation, histone modification, and non-coding RNA-associated gene silencing [145, 146]. SCFAs have pleiotropic effects, play essential roles in numerous molecular biological processes, and can also have effects on tissues and organs beyond the gut through their circulation in the blood [147]. Metabolites of the maternal gut microbiome, such as SCFAs, can regulate the development of the fetal ENS [138]. It has been suggested that epigenetic regulation is a central mechanism by which the environment influences mammalian gene expression in health and disease [148]. SCFAs are essential epigenetic regulators throughout the body and also in the gut [149, 150]. SCFAs, such as butyrate, are inhibitors of histone deacetylases, which, in turn, leads to chromatin changes that are generally associated with increased expression of target genes [151]. Yang *et al.* [152] proposed that SCFAs may control the formation of the ENS by increasing the growth rate of human neural progenitor cells *via* influencing the expression of neurogenesis, proliferation, and apoptosis-related genes. Experiments by Kimura *et al.* [153] suggest that SCFAs such as butyrate and propionate can regulate embryonic development in mice during pregnancy by regulating gene expression of Gpr41 and Gpr43 through epigenetic modifications (Gpr41 and Gpr43 are a pair of mammalian G protein-coupled receptors (GPCRs)).

## 10 SUMMARY WITH HYPOTHESIS

Various studies support the notion that adult health can be influenced by early-life adversity [154]. The fetal programming hypothesis, also known as Barker’s hypothesis, suggests that prenatal conditions can contribute to an individual’s susceptibility to chronic diseases in adulthood, as well as have an impact on offspring ontogenetic vulnerability and mental health outcomes [155-157].

During pregnancy, both psychological and physiological stressors can have a significant impact on maternal and fetal well-being. These stressors include maternal anxiety, depression, and prenatal malnutrition, as well as life events such as trauma, loss, natural disasters, and pathogenic infections [125, 138-140]. Such stressors can disrupt the HPA stress axis and lead to behavioral or cognitive deficits. Maternal infection, anxiety, or depression are associated with increased levels of pro-inflammatory cytokines, including TNF-α, IL-1β, and IL-6, not only in the maternal circulation but also in the fetal circulation and CNS [139-141]. In addition, maternal psychological stress can produce dysbiosis and change the microbiota and microbiota-derived metabolites, such as SCFAs, in the offspring, as well as affect the development of fetal MGBA and impair the permeability of the fetal gut [132, 133, 134, 141]. Furthermore, stressors during pregnancy have been widely linked to epigenetic changes that play a crucial role in determining maternal and offspring health, potentially predisposing individuals to psychiatric problems during childhood [158-160].

### 10.1 Further Developing our Previously Presented Hypothesis

Previously, we presented a novel hypothesis [8] that suggested that during critical developmental periods (*e.g*., prenatal, perinatal, postnatal, and early childhood stages), epigenetically inherited or psychologically induced stressors like maternal separation, maternal depression, or anxiety have the potential to generate stress-induced long-term epigenetic implicit memories (SLEIM) within the fetal ENS. These stress-induced memories may endure within the ENS throughout an individual’s lifetime.

Eventually, SLEIM from within the ENS can cause mild disruption of the intestinal homeostatic processes and transmit these signals to the brain. Although these signals may not be potent enough to cause organic changes, they can lead to symptoms such as abdominal pain, bloating, and alterations in bowel habits, including diarrhea, constipation, or a combination of both. Additionally, SLEIM from the ENS can be communicated to the CNS *via* the MGBA, resulting in dysregulation of affective and pain processing within the CNS [9-14]. Consequently, perturbed cortical-limbic circuits contribute to heightened subjective pain sensitivity (somatization) and the experience of negative emotions. Despite the efforts of the CNS to mitigate the impact of the SLEIM signals from the ENS, the ENS continues to transmit these signals to the CNS through the MGBA. The CNS must cope with the influx of SLEIM signals from the gut. In doing so, it activates stress response systems such as the HPA axis and the immune system, thereby influencing intestinal processes. This establishes a self-perpetuating cycle, commonly referred to as a “vicious cycle,” between the ENS and the CNS.

**Fig. (1):**
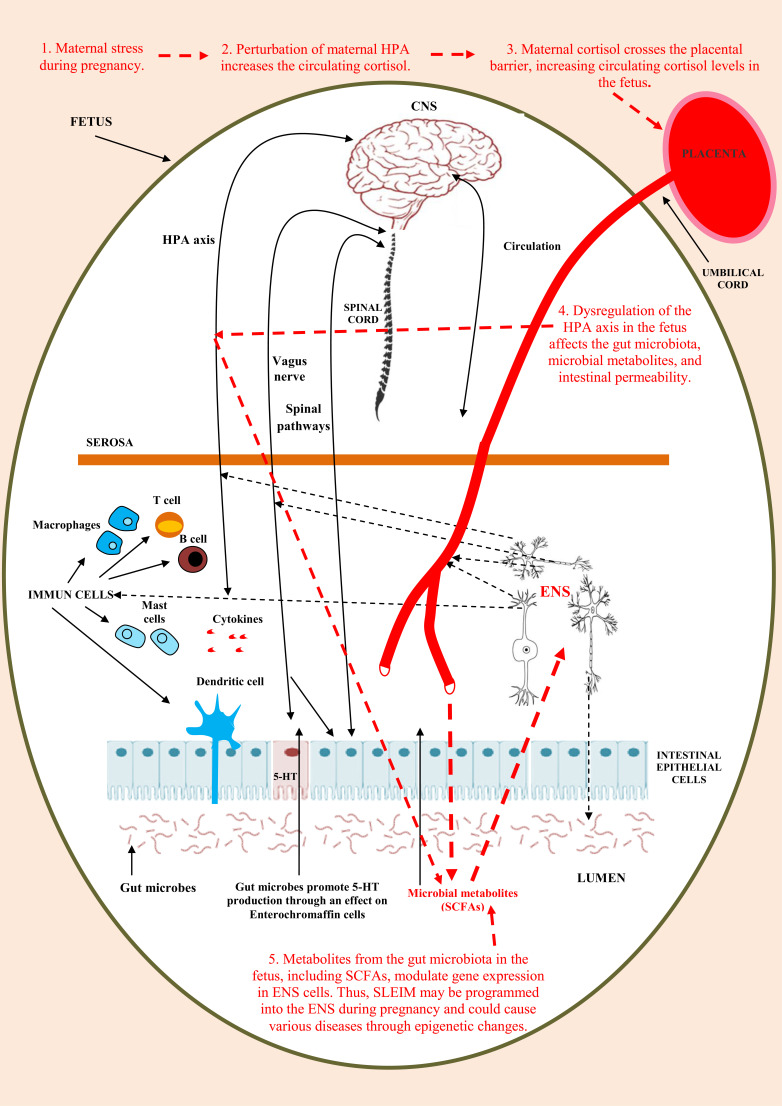
The parts and lines marked in red represent our simplified hypothesis about how stress-induced long-term epigenetic memory (SLEIM) may be programmed into the fetal ENS during pregnancy. The other processes in Figure 1 indicate complex processes of the intestine that could also play various roles in these processes. See a more detailed description in Section 10.1. This figure is adapted with multiple modifications from our open access paper: Császár-Nagy N, Bókkon I. Hypnotherapy and IBS: Implicit and simple stress memory in ENS? Heliyon 2023; 9(1): e12751.

In the present paper, we further develop our previously presented hypothesis and demonstrate how various stresses may produce stress-induced long-term epigenetic memory (SLEIM) in the fetal ENS (Fig. **1**). ENS has a fundamental function as an integrating hub for controlling gastrointestinal physiology [105]. In addition, epigenetic regulations also play an essential role in the functioning of the ENS [120, 122].

As we have seen above, maternal prenatal stress induces a dysregulation of the HPA axis that increases circulating cortisol levels, which can affect the maternal gut microbiota [138]. Maternal cortisol can cross the placental barrier, increase circulating levels in the fetus, and lead to dysregulation of the fetal HPA axis, affecting the gut microbiota, microbial metabolites, and intestinal permeability, among others [139, 140]. In addition, cortisol receptors are expressed on various gut cells, including epithelial cells, immune cells, enteroendocrine cells, and enteric neurons, indicating a direct effect of cortisol on both maternal and fetal gut functions [139].

SCFAs are the major metabolites produced by the microbiota [150] that can control the cortisol response to psychosocial stress [151]. SCFAs can regulate several mechanisms along the MGBA through direct and indirect processes and epigenetic signaling [145]. SCFAs can also regulate the development of the fetal ENS [161]. The metabolites of the gut microbiome, including SCFAs, can affect various diseases by inducing epigenetic changes through DNA methylation, histone modification, and non-coding RNA-associated gene silencing [145, 162, 163].

Finally, one could argue that we are born germ-free. However, studies support the evidence in utero transmission of microbes from mother to infant [163-166]. In addition, maternal metabolites pass from the gut lumen to the circulation, access the fetus through the placenta, reach fetal circulation, and provide the nutrients required for fetal growth and development [167]. So, regardless of the microbiota that could be present in the uterus, the metabolites of the mother's gut microbiota give energy, nutrients, vitamins, folate, choline, betaine, and SCFAs that provide nutritional programming for fetal growth and development through neurotransmitters and epigenetic mechanisms [138].

These mentioned processes suggest that during pregnancy, SLEIM may be programmed into the ENS of the fetus and could persist throughout life, which may play an important role in the development and persistence of IBS.

If our outlined hypothesis can be further confirmed, it may lead to the development of new neuropharmacological and other therapeutic strategies in the future concerning IBS.

## LIST OF ABBREVIATIONS

ANS: Autonomic Nervous System
CH_4_: Methane
CNS: Central Nervous System
CO_2_: Carbon Dioxide
CpG sites: *Cytosine-phosphate-guanine* Sites
DNA: *Deoxyribonucleic Acid*
DNMT: *DNA Methyltransferase*
Dnmt1: DNA Methyltransferase 1
dnmt1: Dnmt1 *Protein* Coding *Gene*
EECs: Enteroendocrine Cells
ENS: Enteric Nervous System
EPC: Enteric Progenitor Cell
GABA: Gamma-aminobutyric Acid
GALT: Tut-associated Lymphoid Tissue
GT: Gastrointestinal Tract
H_2_: Hydrogen
H_2_S: Hydrogen Sulfide
HPA axis: Hypothalamic-Pituitary-Adrenal Axis
HSCR: Hirschsprung Disease
IBD: Inflammatory Bowel Diseases
IBS: Irritable Bowel Syndrome
IL-1β: Interleukin-1 Beta
IL-6: Interleukin 6
MGBA: Microbiota-gut-brain Axis
MORs: µ-opioid Receptors
NCC: Neural Crest Cell
NO: Nitric Oxide
PNS: Parasympathetic Nervous System
PRC1: Polycomb Repressive Complex 1
PRC2: Polycomb Repressive Complex 2
Ring1b: Enzymatic Component of the PRC1 Complex
*rnf2*: Ring Finger Protein 2 Protein Coding *Gene*
SCFAs: Short-chain Fatty Acids
SLEIM: Stress-induced Long-term Epigenetic Memory
SNS: Sympathetic Nervous System
SSRI: Selective Serotonin Reuptake Inhibitors
TNF-α: Tumour Necrosis Factor-alpha
Uhrf1: Ubiquitin-like Protein Containing PHD and RING Finger Domains 1
uhrf1: Uhrf1 *Protein* Coding *Gene*
VN: Vagus Nerve
